# Characterization of Flow with a V-Shaped NMR Sensor

**DOI:** 10.3390/s24196163

**Published:** 2024-09-24

**Authors:** Eric Schmid, Tim Oliver Pertzel, Hermann Nirschl, Gisela Guthausen

**Affiliations:** 1 Institute of Mechanical Process Engineering and Mechanics, Karlsruhe Institute of Technology, 76131 Karlsruhe, Germany; 2 Chair of Water Chemistry and Water Technology, Engler-Bunte-Institut, Karlsruhe Institute of Technology, 76131 Karlsruhe, Germany

**Keywords:** low-field NMR, inline process monitoring, NMR sensor, battery slurries, flow NMR, MRI

## Abstract

Quality control in a production plant shows its maximum potential in the form of inline measurements. Defects and imperfections can be detected early and directly, and waste and costs can be reduced. Nuclear Magnetic Resonance offers a wide range of applications but requires dedicated adaptation to the respective process and material conditions. A V-shaped low-field NMR sensor was developed for non-invasive inline measurements on anode slurries in a battery production plant. In battery production, inline monitoring of the quality of anode slurries is demanded, offering the possibility of predictive control of the following process steps. Methods of low-field NMR to determine flow properties were adapted to the desired application. Further, magnetic resonance imaging measurements were made to determine the flow properties of model substances and anode slurries, thus providing verification. The sensor measurements show the ability to measure the flow behavior of, amongst other fluids, anode slurries in a form suitable for inline quality control in a battery production plant.

## 1. Introduction

Inline analytics is a valuable component in a processing plant. Continuously recorded data offer comprehensive quality control whilst manufacturing a product and in autonomous process control. In the production of lithium-ion battery anodes, between the process steps of wet mixing the raw components in an extruder and coating the current collectors with the active material, questions arise about the quality of the paste as an intermediate product. Quality characteristics include the chemical composition of the slurry, the occurrence of gas inclusions, separation processes, and rheological properties. The quality of the paste directly influences the quality of the electrode and in consequence is responsible for the final performance of the battery [1]. Furthermore, an efficient inline analytic technology enables reductions in waste and thus cost reduction and saving of resources [2,3].

Nuclear Magnetic Resonance (NMR) is an analytical technique that has been established in various applications. Numerous examples of NMR in industrial quality control [4,5] make use of the non-invasive property of NMR, apart from the chemical structure elucidation via NMR spectroscopy. Low-field (LF) NMR techniques have, compared to the high-field variant, the advantages of requiring less space, lower operating costs due to the use of permanent magnets, and the option of adapting the instrument specifically to the intended application, which maximizes the gain of relevant information [6,7,8,9,10]. However, the low-field variants often have technical constraints regarding *B*_1_ and *B*_0_ magnetic fields and their homogeneity, which need a detailed theoretical description of the NMR signal considering all coherences in a pulse sequence [11]. In addition to offline applications, there are also some examples of inline flow sensors. Flow measurements open up the possibility of determining rheological parameters by NMR. In particular, the sensitivity of the NMR signal phase to flow is explored, for example, in multi-echo pulse sequences. This approach is especially promising when dealing with a constant magnetic field gradient to determine the flow velocity fields [12,13,14]. For example, the velocity field in a tube flow leads to a distribution in the signal phases according to the Hagen–Poiseuille law for Newtonian fluids. With this and more generalized approaches, the flow behavior index of a flowing sample can be determined inline, providing rheological information about the liquid [12,13,15].

The LF NMR methods for measuring flow known in the literature are applied to the inline-capable V-shaped NMR sensor [16,17]. The sensitivity of the measurements to quality parameters in battery anode production, including chemical composition, sedimentation, and gas inclusions, was already proven [17,18]. Furthermore, the basic sensitivity to flow could be demonstrated [18]. Known LF flow measurement methods [12,13,14] were explored to use the V-sensor for the desired application of inline quality control in anode slurry production. The results are verified by flow measurements on reference substances using established high-field MRI (magnetic resonance imaging). The inline measurements of flow velocity fields and the flow behavior index open up the possibility of measuring the rheological behavior of the anode slurries inline and thus increase the information gain. The flow behavior is a direct indicator of the slurry quality and non-invasive rheological measurements are a promising component in process control in battery production. In connection with the sensitivities already shown, the NMR sensor has the potential to combine several conventional analysis methods in one device. It should be mentioned that the method is not limited to anode slurries, but can be adapted to other types of fluidic flow, provided that the transverse relaxation is sufficiently long for an appropriate sensitivity to the detection of phase shifts.

## 2. Materials and Methods

### 2.1. Methodological Background

The power law model of Ostwald and de Waele describes the mathematical relation between the shear stress τ, the flow consistency index *k*, and the shear rate $\dot{\gamma }$ with a dimensionless exponent *n*, the flow behavior index (Equation (1)) [19,20].

(1) $$\tau =k{\dot{\gamma }}^{n}$$

*n* reflects the flow character of the investigated fluid. For Newtonian fluids, *n* = 1 applies. Pseudoplastic, shear-thinning behavior leads to *n* < 1, whereas for dilatant, shear-thickening fluids, *n* > 1. The determination of *n* is therefore desired for process monitoring to generate rheological information about the sample and thus to assess the quality.

A fully developed axial flow velocity profile in a tube as a function of the radial coordinate *r* is given by the mean flow velocity *v*_mean_, the tube radius *R* and *n* (Equation (2)) [19,21].

(2) $$v\left(r\right)=v_{mean}\frac{3n+1}{n+1}\left[1-{\left(\frac{r}{R}\right)}^{1+\frac{1}{n}}\right]$$

*r* = 0 is the center of the tube. NMR echo intensities *I* are described by complex numbers with a real part *Re* and imaginary part *Im*. The magnitude *M* of the signal is given by Equation (3).

(3) $$M=\sqrt{{I_{Re}}^{2}+{I_{Im}}^{2}}$$

The signal phase ϕ is defined by the well-known trigonometric rule (Equation (4)):

(4) $$\phi =arctan\left(\frac{I_{Im}}{I_{Re}}\right)$$

The phase shift between the NMR signal of a static sample at *v* = 0 and a flowing sample (*v* ≠ 0) is given by Equation (5):

(5) $$\Delta \phi =\phi_{v}-\phi_{v=0}$$

CPMG (according to Carr, Purcell, Meiboom and Gill [22,23]) measurements on a flowing sample lead to a phase shift only in the odd echoes of the echo train, for example [13,24,25,26,27]. The prerequisite is a magnetic field gradient *G* in the direction of flow. The phase of an odd echo is given by Equation (6), with the gyromagnetic ratio γ, the echo time τ_e_ and *v*_mean_. Please note that τ_e_ corresponds to the time between two refocusing pulses in the CPMG pulse sequence. *v*_mean_ can be determined from the measured signal phases by rearranging the equation.

(6) $$\phi_{odd}=\frac{1}{4}\gamma G{\tau_{e}}^{2}v_{mean}$$

The mean flow velocity can therefore be determined using the CPMG pulse sequence also in the static magnetic field gradient *G* of the V-shaped sensor. Please note that the product of squared echo time and *G* enter the equation; accordingly, the timing of the CPMG sequence and the static gradient play a significant role for lower and upper limits of detection as well as for the statistical significance of the measurements.

It is also possible to measure the flow behavior index *n* via the analysis of the signal magnitudes. Flow profiles lead to a distribution of flow velocities in an integral measurement and, with that, to a distribution of the phase accumulation at the odd echoes of the CPMG echo train [12]. The accumulation influences the signal magnitude. The magnitudes can be modeled using a gamma distribution function Γ and the flow behavior index. The normalized magnitude as a function of τ_e_ shows damped oscillatory behavior. Equation (7) allows *n* to be determined in a non-linear least square fit to the data [12,13]:

(7) $$M=\frac{2n}{n+1}{\left(\frac{\gamma G\left(3n+1\right){\tau_{e}}^{2}v_{mean}}{4\left(n+1\right)}\right)}^{-\frac{2n}{n+1}}*\;\sqrt{\begin{array}{c} \left(\Gamma \left(\frac{2n}{n+1}\right)-\Gamma \left[\frac{2n}{n+1},-\frac{i\gamma G\left(3n+1\right){\tau_{e}}^{2}v_{mean}}{4(n+1)}\right]\right)\left(\Gamma \left(\frac{2n}{n+1}\right)-\Gamma \left[\frac{2n}{n+1},\frac{i\gamma G\left(3n+1\right){\tau_{e}}^{2}v_{mean}}{4(n+1)}\right]\right) \end{array}}$$

### 2.2. Short Description of the NMR Sensor

**Figure 1:**
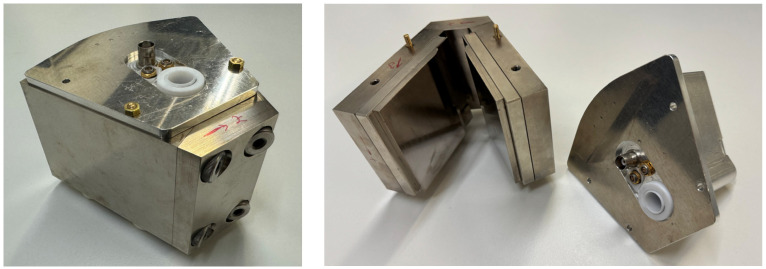
**Left**: Picture of the V-sensor with the white PTFE cylinder in which the tube is positioned. **Right**: The V-shaped magnet arrangement (left side) and the closed probe for volumetric measurements of samples with an outer diameter up to 12 mm (right side).

The NMR sensor in this work is a palm-sized low-field NMR sensor with a V-shaped NdFeB magnet arrangement; the ^1^H Larmor frequency is 22.1 MHz (Figure 1). In addition to the closed radio frequency probes for volumetric measurements on samples with a diameter of 12 and 42 mm [16], an open geometry with a bent figure-8 surface coil is available, which allows measurements on a tube by placing the sensor on it [17,18]. A closed 12 mm probe was chosen here for integral measurements of the flow fields. A commercial electronic unit was used for control of the sensor and pulse generation (Bruker ‘the minispec’ ND-series). The tube was positioned in a PTFE cylinder in the center of a solenoidal coil. The V-shaped arrangement of the magnet unit amounts to a mean effective magnetic field gradient of *G*_eff_ = 0.69 T/m of the static magnetic field *B*_0_ for a 10 mm sample, providing, in principle, the sensitivity to flow. This value was determined by diffusion measurements in [16] with a distribution width of 0.35 T/m. When calculating the gradient by correlation of the calculated mean flow velocities from NMR with volumetrically determined mean flow velocities of tap water, a value of *G*_eff_ = 0.22 T/m was obtained. Only the gradient in the flow direction needs to be taken into account. For further information on the design of the sensor, we refer to [16,17].

### 2.3. Experimental Setup of the Flow Measurements

**Figure 2:**
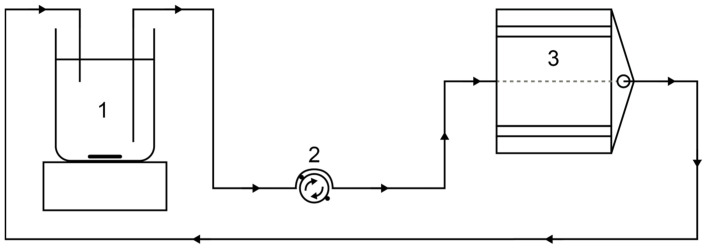
Experimental setup of the flow measurements with the NMR sensor. The sample was in a beaker (1) and stirred continuously with a magnetic stirrer to avoid sedimentation. A peristaltic pump (2) generates flow in a tube through the NMR sensor (3). The sample is returned to the beaker for a closed circuit.

A Poly(methyl methacrylate) (PMMA) tube with an inner diameter of 8 mm and a wall thickness of 0.35 mm was positioned in the probe of the sensor and fluidically connected with the experimental setup for flow measurements (Figure 2).

The sample is continuously stirred in a beaker with a magnetic stirrer in order to prevent sedimentation. A peristaltic pump Ismatec IPC-N-8 V3.01 (Ismatec SA, Glattbrugg-Zürich, Switzerland) was used for pumping the sample through the tubing. The pump was equipped with eight parallel channels that were connected to a single channel after the pump to provide sufficiently large flow velocities during the measurement. After passing the NMR sensor, the sample was fed back to the beaker to allow for continuous experiments in a closed loop (Figure 2).

**Figure 3:**
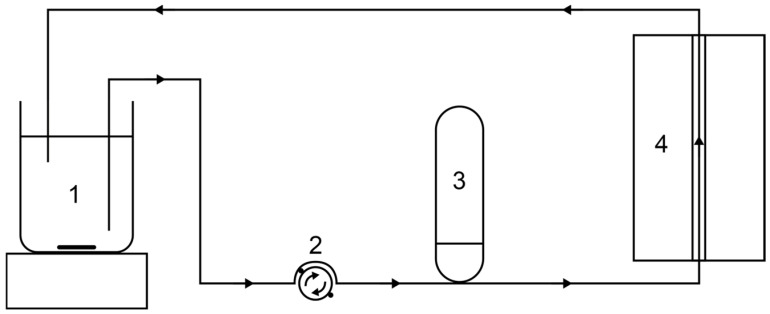
Experimental setup of the flow MRI measurements. The sample is stirred in a beaker (1) and pumped with the peristaltic pump (2) through the tomograph (4). A pressure compensation tank (3) reduced pulsation.

A 200 MHz Bruker Avance III HD spectrometer (Bruker BioSpin GmbH, Ettlingen, Germany) with a microimaging setup was used for the MRI measurements. A polyurethane tube with an outer diameter of 10 mm and an inner diameter of 7 mm was passed through the ^1^H birdcage probe. A Watson Marlow 323S peristaltic pump (Watson-Marlow Ltd., Falmouth, England) was used to pump the sample through the magnet’s bore along a height of 1.5 m. A pressure compensation tank was added to the fluidic circuit before passing the magnet in order to reduce flow pulsation due to the Watson Marlow pump (Figure 3).

To validate the flow velocities measured via the V-sensor and MRI, the mean flow velocities were also measured volumetrically. The volume was measured with measuring cylinders over a defined time, allowing the mean flow velocity *v*_mean,vol_ to be calculated.

### 2.4. Composition of the Samples

Model systems and anode slurries were investigated (Table 1). Tap water was chosen as a model substance for a Newtonian fluid. Anode slurries are typically water-based. Other ingredients are graphite powder as active material, carboxymethylcellulose (CMC) and styrene-butadiene rubber (SBR) as binders, and carbon black (CB) as an electrically conducting additive. The anode slurries were produced with an extruder on a pilot plant scale. Aqueous CMC solutions were used in different concentrations as a model system for shear-thinning fluids. Further, a corn starch suspension is an example of a shear-thickening liquid. The same applies to a suspension of graphite particles in water. The CMC powder of Sigma Aldrich is characterized by a molecular weight of 700,000 u and a degree of substitution between 0.8 and 0.95.

### 2.5. Measurement Parameters

The spatially resolved velocity measurements were made with the FLOWMAP pulse sequence [28,29,30,31]. Measurements were made on tap water, CMC solutions and diluted anode slurry with the measurement parameters listed in Table 2. Due to the different NMR properties of the samples, the parameters needed a specific adaptation.

The measurements with the V-shaped NMR sensor were made with the CPMG pulse sequence in its low-field version. Whole echo trains were acquired after a single excitation due to the fast *T*_2_* relaxation which is different from the CPMG implementation in the homogeneous magnetic fields in most high-field NMR instruments. Measurements of the transverse relaxation via decay of the magnetization’s magnitude were made with the CPMG pulse sequence. Complex magnetization decays were measured to deduce the velocity information. Parameters were adapted to the sample characteristics, so that the anode slurry and the graphite-in-water suspension were measured with specific parameters (Table 3).

### 2.6. Data Analysis

MRI: The FLOWMAP pulse sequence provides two-dimensional spatially resolved velocity maps. Flow profiles that can be described with the power law model were calculated [32]. For the radially symmetric cross section of a cylindric tube, concentric rings with a width of a single pixel were defined. The diameter of the rings is reduced with each step in the calculation. The mean flow velocity was calculated in each ring, which provides one half of a flow profile. This can then be described with the flow models.

V-sensor: The raw data in the form of complex magnetization decays were modeled with fit models according to the sample’s properties. A biexponential model was used for binary mixtures, whereas the magnetization decays of more complex samples, like battery anode slurries, can more accurately be described with a distribution model like the gamma model [33,34]. Equations (3)–(7) were used for the determination of *v*_mean_ and *n*.

## 3. Results

### 3.1. Reference Measurements with Established MRI Methods

**Figure 4:**
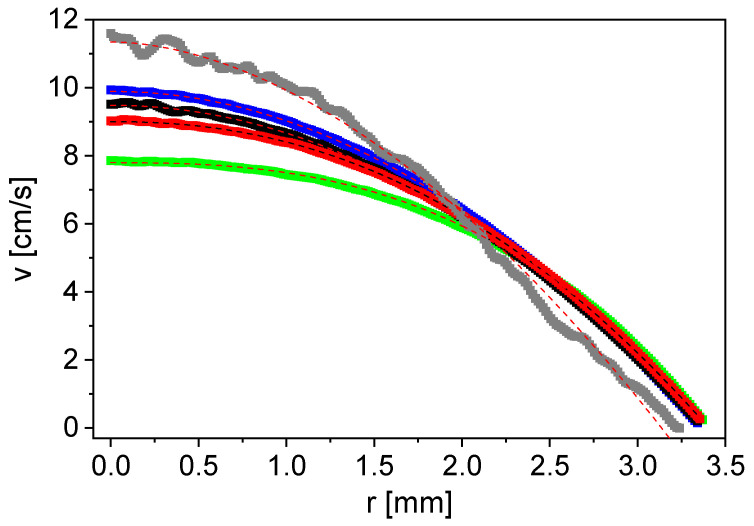
Flow velocity as a function of the radial coordinate, including the power law fits, for water (■), 0.4% w/w CMC in water (■), 0.8% w/w CMC in water (■), 1% w/w CMC in water (■) and anode slurry with 10.4% w/w solids content (■) for *v*_mean,vol_ = 4.8 cm/s. The flow profiles reflect the rheologic properties.

MRI was performed to characterize the samples regarding their NMR and flow properties, and to find suitable measurement parameters. Measurements on non-flowing samples (*v* = 0 cm/s) were made with the pulse sequence MSME for parameter optimization for all samples. The slurry sample stands out from the other samples due to its smaller signal to noise ratio, but also regarding the much faster transverse relaxation. The two-dimensional flow velocity fields were measured with the FLOWMAP pulse sequence and processed with radial averaging [32]. The flow velocity as a function of the tube radius differs for water, aqueous CMC solutions and an anode slurry, measured at *v*_mean,vol_ = 4.8 cm/s (Figure 4).

The flow velocity profile of water shows Newtonian behavior as expected (Figure 4). The maximum flow velocity decreases with increasing CMC concentration for the CMC solutions. The radial change in velocity in the center of the tube becomes increasingly smaller; a flattening of the flow profile is observed. This is typical for a shear-thinning fluid with a flow behavior index *n* < 1. The measurement of the diluted anode slurry shows a shear-thickening character, which does not meet the initial expectations of CMC dominating the rheologic behavior of the slurry. However, properties similar to a particle-loaded liquid and thus to shear thickening are reasonable at small CMC concentrations, as in the present case [35]. The flow profiles (Figure 4) were modeled with the power law fit (Equation (2)) to determine the flow behavior index *n* (Table 4).

As expected, water shows Newtonian behavior with *n* = 1. The same value is obtained for the smallest CMC concentration of 0.4% w/w. Nevertheless, a flattening of the flow profile is already recognizable (Figure 4). *n* becomes smaller when increasing the CMC concentration, which describes the increasing shear-thinning character. The shear-thickening behavior of the anode slurry results in *n* = 1.2 (Table 4).

**Figure 5:**
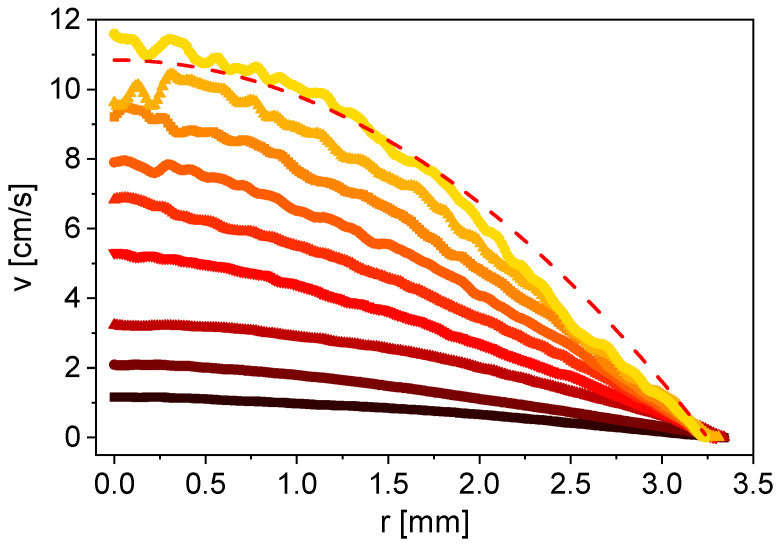
Flow velocity profiles of the diluted anode slurry with a solids content of 10.4% w/w for *v*_mean,vol_ of 0.4 cm/s (■), 0.8 cm/s (●), 1.3 cm/s (▲), 1.9 cm/s (▼), 2.6 cm/s (▲), 3.1 cm/s (●), 3.6 cm/s (■), 4.3 cm/s (▲) and 4.8 cm/s (■). Additionally, a calculated Hagen–Poiseuille flow profile (**--**) to provide evidence for the shear-thickening character of the anode slurry.

In order to investigate the flow behavior of the anode slurry in more detail, spatially resolved velocity measurements were carried out at various mean flow velocities between *v*_mean,vol_ = 0.4 and 4.8 cm/s (Figure 5). The comparison with an ideal Hagen–Poiseuille profile (*v*_mean,vol_ = 4.8 cm/s) illustrates the shear-thickening behavior, which is independent of the mean flow velocity.

### 3.2. Transverse Relaxation at 22 MHz

The effective transverse relaxation given by the rate *R*_2,eff_ of a sample is an important basis for the selection of suitable measurement parameters and the interpretation of the measurement results. CPMG magnetization decays with the NMR sensor were measured on non-flowing samples and modeled with a gamma model (tap water, anode slurry, 30% w/w graphite in water and 40% w/w corn starch in water) and a biexponential fit equation for the aqueous CMC solutions (Table 5).

Tap water has the smallest *R*_2,mean,eff_ as expected, whereas the anode slurry shows the largest value. Two relaxation times result from the biexponential fit model for the CMC-in-water solutions with the fractions *x*_1_ and *x*_2_.

### 3.3. Measurement of Flow Velocities by NMR Signal Phase Shifts

**Figure 6:**
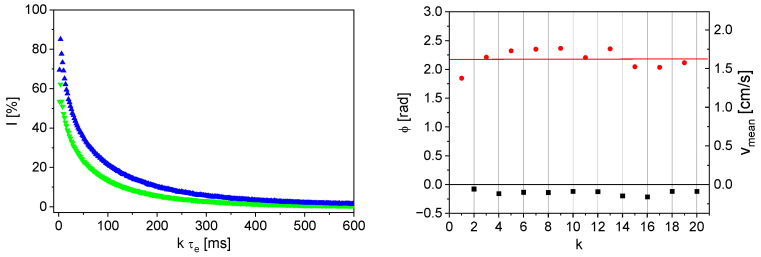
**Left**: Exemplary real (▼) and imaginary parts (▲) of a complex signal decay from a CPMG measurement on tap water. **Right**: Calculated phase shifts and mean flow velocities for even (■) and odd (●) echoes of a CPMG measurement on tap water with a volumetrically determined *v*_mean,vol_ of 1.62 cm/s (red line) and τ_e_ = 2.8 ms. As expected, only the odd echoes are sensitive to flow.

The feasibility of the phase shift approach was first demonstrated on tap water. The phase shifts between the echoes of experiments with tap water at *v*_mean,vol_ = 1.62 cm/s and *v*_mean_ = 0 cm/s (Equation (5)) were calculated from the complex signal decays (Figure 6, left). *v*_mean_ then follows according to Equation (6) (Figure 6, right).

**Figure 7:**
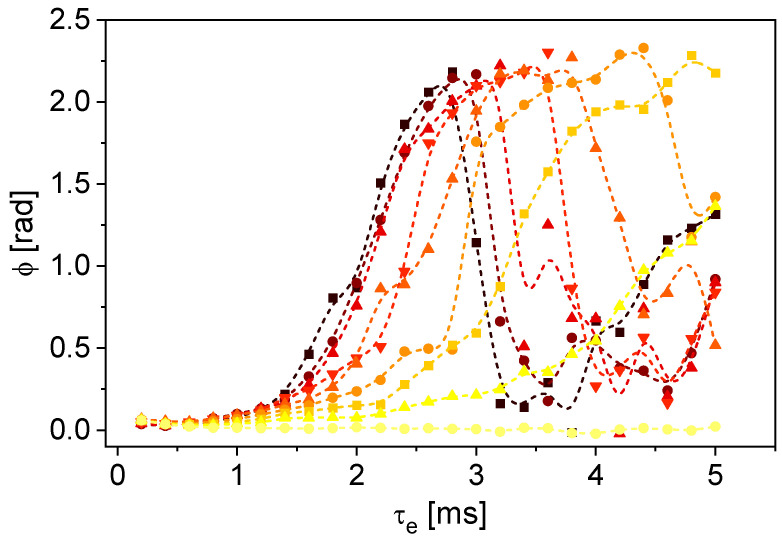
Average phase shifts of the first 10 odd echoes as a function of τ_e_ for different *v*_mean,vol_ between 0 cm/s (●), 0.19 cm/s (▲), 0.39 cm/s (■), 0.57 cm/s (●), 0.78 cm/s (▲), 0.97 cm/s (▼), 1.2 cm/s (▲), 1.41 cm/s (●) and 1.62 cm/s (■). B-splines are shown as guides to the eyes. The phase shifts for different flow velocities have a maximum at a specific τ_e_. Smaller velocities lead to a maximum at higher τ_e_.

The odd echoes are sensitive to flow, whereas the even echoes are not (Figure 6, right). The calculated velocities are in the same range as the volumetrically determined *v*_mean,vol_ of 1.62 cm/s. The fluctuations in the values depend on the echo number *k*. In particular, a systematic deviation of approximately 20% is observed for the first echo, which is due to the technical realization of magnet and radio frequency unit in the V-shaped sensor. At large numbers of echoes, the statistical error increases due to the more and more limited signal-to-noise ratio along the magnetization decay. Under the given conditions, the method is well suited for measuring the mean flow velocity, which deviates from *v*_mean,vol_ by a maximum of 9% from the second echo. It became evident that a suitable value for the echo time is necessary for the flow sensitivity of the odd echoes, whereby the used echo time of 2.8 ms is the optimized value for *v*_mean,vol_ = 1.62 cm/s. The phase shifts were measured as a function of τ_e_ for flow velocities *v*_mean,vol_ ∈ [0, 1.62] cm/s (Figure 7). The measurement at *v*_mean,vol_ = 0 cm/s was made last; the reference measurement for calculating the phase shift for all velocities was the first measurement in the series. The Reynolds number for the water flow with *v*_mean,vol_ = 1.62 cm/s is approximately 129 and thus in the laminar flow range.

The phase shift reaches a maximum as a function of τ_e_, which shifts towards smaller τ_e_ for larger velocities (Figure 7). To minimize statistical errors, the measurement should be performed at an echo time that leads to maximum phase shift—which is limited by the transverse relaxation on the other hand. τ_e_ was specifically chosen for each velocity at which the phase shift is maximum.

**Figure 8:**
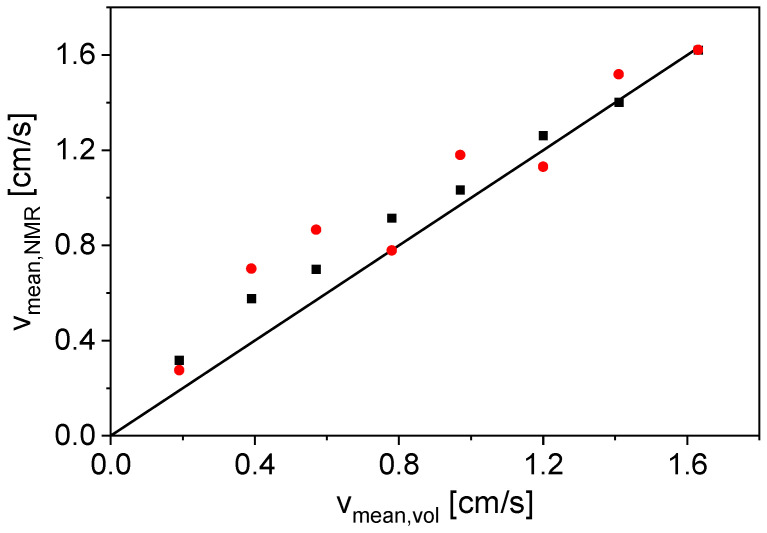
Mean flow velocities of tap water calculated from the phase shifts with a static measurement as a reference (■) and the average of the first 10 even echoes as reference (●) as a function of *v*_mean,vol_. A correlation line (**–**) was added as a guide to the eyes.

Measurements at *v*_mean,vol_ = 0 cm/s were used as a reference for the phase shift calculation (Figure 6 and Figure 7). However, it is also possible to use the phases of the even echoes at *v*_mean,vol_ ≠ 0 cm/s as a phase reference; here, an average value of the first 10 even echoes was used. This makes an additional measurement with *v*_mean,vol_ = 0 cm/s unnecessary. To show the comparability of the two referencing methods, the mean flow velocities were calculated via both methods and correlated with *v*_mean,vol_ (Figure 8).

Both referencing methods are suitable for measuring *v*_mean,NMR_ of tap water in the investigated velocity range (Figure 8). The deviation from the angle bisector is slightly larger for the measurements with the even echoes as a phase reference (79% maximum deviation from *v*_mean,vol_), but the reference measurement at *v*_mean,vol_ = 0 cm/s is not necessary in the measurement procedure, which is an advantage for the application of the method in process control. Furthermore, changes in temperature and pressure between a flow measurement and the reference measurement would not interfere and lead to reduced accuracy, if no reference measurement is needed.

**Figure 9:**
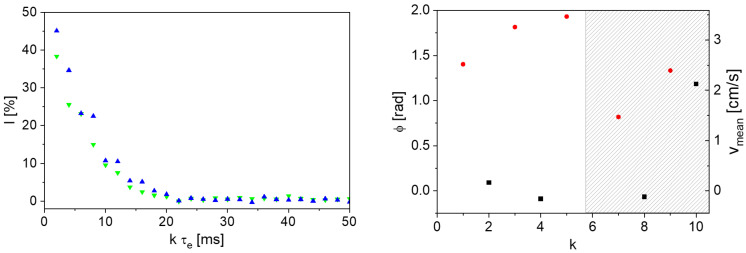
**Left**: Complex CPMG magnetization decay with *v*_mean,vol_ = 0 cm/s on an anode slurry (▼: real part, ▲: imaginary part) with τ_e_ = 2 ms. **Right**: Phase shift and mean flow velocity for the first 10 echoes for a measurement on anode slurry with τ_e_ = 1.8 ms and *v*_mean,vol_ = 3.04 cm/s (■: even echoes, ●: odd echoes). The shaded area indicates the echoes with a signal intensity at noise level.

To investigate the suitability of the adapted method on the application of the sensor in a battery anode manufacturing process, flow measurements were also made on an anode slurry with a solids content of 45% w/w (Figure 9). The transverse relaxation for the anode slurry is much faster than for tap water (Table 5). Measurements of the phase shift as a function of τ_e_ (analogous to Figure 6) have shown a maximum phase shift at τ_e_ = 1.8 ms in the range of *T*_2,mean,eff_ of the anode slurry for *v*_mean,vol_ = 3.04 cm/s; laminar flow can be assumed. A CPMG measurement with τ_e_ = 2 ms shows that only the first eight echoes show a signal intensity above the noise level (Figure 9, left).

**Figure 10:**
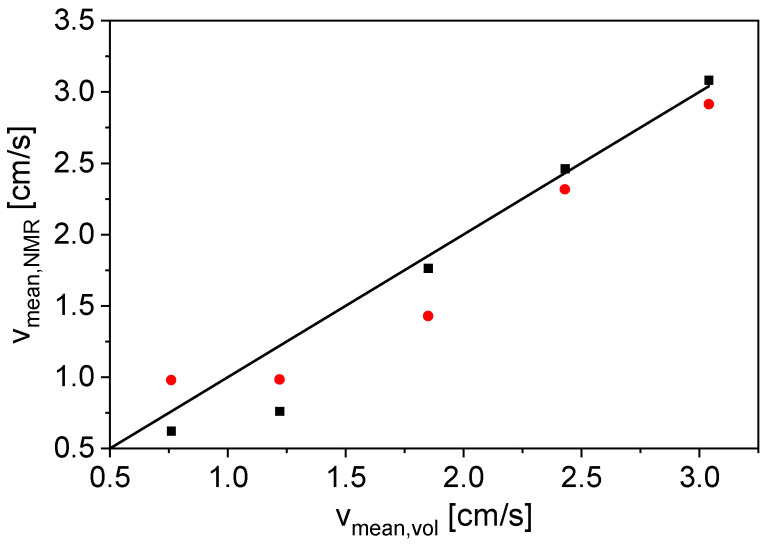
Calculated mean flow velocities with a measurement at *v*_mean,vol_ = 0 cm/s as a reference (■) and the first two even echoes as a reference (●) as a function of the volumetrically measured *v*_mean,vol_ for an anode slurry. The correlation line (**–**) was added as guide to the eyes.

The measurements on the anode slurry also show the sensitivity of the odd echoes to flow (Figure 9, right). Due to the fast transverse relaxation in combination with the necessity to measure with relatively large τ_e_, only the first five echoes have a sufficient signal intensity. A measurement with *v*_mean,vol_ = 0 cm/s was used as phase reference for this measurement (Figure 9, right). The two referencing methods were compared to investigate whether referencing with the even echoes is still suitable for an application in inline quality control of anode slurries (Figure 10).

The results demonstrate that both referencing methods can be used for flow measurements on anode slurries. The mean flow velocities are provided with sufficient accuracy and a maximum deviation of 38% from *v*_mean,vol_. Better accuracy can be achieved by reducing the receiver dead time through further optimization of the NMR hardware. This facilitates the measurement of samples with fast transverse relaxation.

### 3.4. Flow Behavior Index from Signal Magnitudes

**Figure 11:**
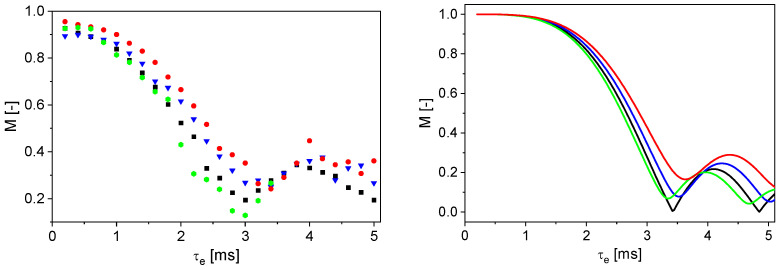
Normalized signal magnitudes as a function of τ_e_. **Left**: Measured magnitudes for tap water (■), 0.4% w/w CMC in water (▼), 1% w/w CMC in water (●), 40% w/w corn starch in water (●). **Right**: Calculated reference values for comparison: *n* = 1 (**–**), *n* = 0.7 (**–**), *n* = 0.5 (**–**), *n* = 1.5 (**–**). Water shows Newtonian behavior. The aqueous CMC solutions have the characteristics of a shear-thinning fluid, whereas corn starch in water shows shear-thickening behavior. The small deviation in the τ_e_ axes is a consequence of the value of the effective magnetic field gradient in the experiments and the simulations.

Various samples with different flow behavior were analyzed at a mean flow velocity of 0.58 cm/s as a function of τ_e_ to investigate the sensitivity to the flow behavior index *n*. The normalized magnitudes *M* of the odd echoes were calculated and plotted against τ_e_ (Figure 11, left). In addition, the theoretically calculated curves are shown for four different *n* values between 0.5 and 1.5 (Figure 11, right). The theoretical values were calculated with *G* = 0.69 T/m to show comparable τ_e_ axes in the diagrams.

**Figure 12:**
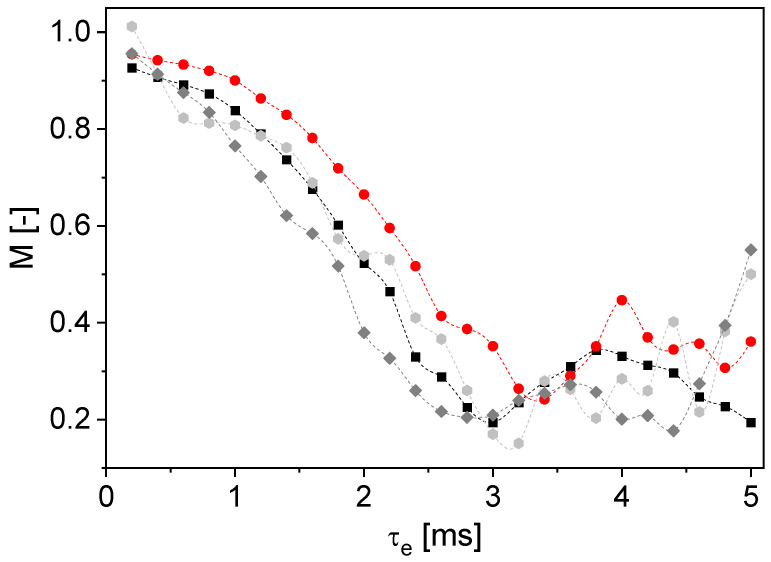
Magnitudes normalized to a measurement at *v*_mean,vol_ = 0 cm/s as a function of τ_e_ for tap water (■), anode slurry with 45% w/w solids content (⬢), 1% w/w CMC in water (●) and 30% w/w graphite in water (◆) for *v*_mean,vol_ = 0.58 cm/s. The graphite-in-water suspension shows shear thickening, whereas the curve for the anode slurry is located between the curve of CMC in water and graphite in water. Splines were added as guides to the eyes.

*n* = 1 leads to a damped oscillation. The measured data do not show *M* = 0 at a specific τ_e_ for *n* = 1, which is in contrast to the mathematical description. This can be explained by the distribution of the magnetic field gradient in the sensitive area of the sensor and the velocity distribution in the tube. Additionally, the measurement accuracy is limited, contributing to a statistical error. *n* < 1 shifts the curve to the right and upwards in comparison to Newtonian behavior (Figure 11, right). The shift is to the left and upwards for *n* > 1. Similar behavior was observed in the measured data (Figure 11, left): a behavior typical for *n* < 1 is visible for the aqueous CMC solutions with two different concentrations; the values of *M*(τ_e_) are shifted to the right and upwards as a result of shear thinning. This finding is in good agreement with the MRI results (Figure 4, Table 4). The aqueous corn starch suspension has a shear-thickening behavior. As expected for *n* > 1, *M*(τ_e_) is shifted to the left. It should be noted that no measured data are displayed for τ_e_ > 3.6 ms because of the fast transverse relaxation of the corn starch suspension (Table 5). The inline determination of *n* with the V-shaped NMR sensor works well for the investigated model substances under the given conditions. The measurements were also performed on an anode slurry with a solids content of 45% w/w and a suspension of graphite particles in water with 30% w/w graphite (Figure 12), where the signal intensities were normalized to that of the corresponding first echo at *v*_mean,vol_ = 0 cm/s. Due to the fast transverse relaxation and the small signal to noise ratio of the anode slurry, a larger number of phases of odd echoes was averaged for small τ_e_.

*M*(τ_e_) shows shear thickening for the graphite suspension typical for particle-loaded liquids (Figure 12). This finding is consistent with the results on the diluted anode slurry in the MRI measurements (Figure 4). *n* < 1 applies to the aqueous CMC solution (1% w/w). The anode slurry shows behavior between the graphite suspension and the aqueous CMC solution, comparable with the Newtonian fluid water. This corresponds to the expectations because the graphite particles in the slurry cause shear thickening, whereas CMC leads to shear-thinning behavior and the gradual transition between the types of rheologic behavior is observed. The normalized signal magnitudes reflect the flow behavior index *n* as a function of τ_e_, including an anode slurry with a technically relevant composition.

## 4. Conclusions

The measurement sensitivity of the V-shaped NMR sensor to flow was investigated. Methods for using the flow-induced NMR signal phase shifts and the determination of the flow behavior index from the signal magnitudes were adapted for use with the V-sensor. The feasibility of CPMG echo train analysis was demonstrated on model substances with different flow behavior. Measurement and data analysis can be applied to aqueous slurries for battery anode production by selecting suitable measurement parameters. The measurement of flow properties significantly expands the V-shaped sensor’s field of application in the area of inline quality control and provides valuable information about the quality of the target substance and the production process. Several quality parameters such as chemical composition, gas inclusion and flow behavior can be determined in parallel within one NMR measurement, and in perspective, invasive or offline analytical methods can be replaced or complemented in a useful way. Besides the application of the sensor in a battery production plant, various other fields of non-invasive inline quality control are conceivable, including cosmetics and pharmaceutical industries and food manufacturers. Future optimization of the sensor hardware and measurement strategies will improve the measurements on samples with fast transverse relaxation and increase the range of possible applications with better accuracy.

## Figures and Tables

**Table 1:** Composition of the samples for flow measurements.

Sample Name	Composition	Measured with
Tap water	Tap water	MRI, V-sensor
0.4% w/w CMC in water	0.4% w/w CMC powder in tap water	MRI, V-sensor
0.8% w/w CMC in water	0.8% w/w CMC powder in tap water	MRI
1% w/w CMC in water	1% w/w CMC powder in tap water	MRI, V-sensor
40% w/w corn starch in water	40% w/w corn starch in tap water	V-sensor
30% w/w graphite in water	30% w/w graphite powder in tap water	V-sensor
Diluted anode slurry	89.6% w/w demineralized water 9.7% w/w graphite 0.39% w/w SBR 0.19% w/w CMC 0.15% w/w CB	MRI
Anode slurry	55% w/w demineralized water 41.85% w/w graphite 1.35% w/w SBR 0.795% w/w CMC 0.595% w/w CB	V-sensor

**Table 2:** Measurement parameters of the spatially resolved velocity measurements using the FLOWMAP pulse sequence.

Parameter	Water, CMC Solutions	Diluted Anode Slurry
Echo time τ_e_ [ms]	10	2.4
Repetition time [ms]	400	400
Flip angle [°]	60	60
Number of averages [-]	3	4
Field of flow [cm/s]	1 … 15	2 … 20
Slice thickness [mm]	2	4
Image size [-]	128 × 128	128 × 128

**Table 3:** Measurement parameters of the flow measurements with the NMR sensor for the different samples.

Parameter	Water, CMC Solutions, Corn Starch Suspension	Anode Slurry, Graphite-in-Water Suspension
Echo time τ_e_ [ms]	0.2 … 5.2	0.2 … 5.2
Number of echoes *k* [-]	500	150
Recycle delay [s]	10	4
Receiver gain [dB]	69	72
Number of averages [-]	8 … 16	16 … 64

**Table 4:** Flow behavior indexes *n* of the samples, calculated from the flow profiles in Figure 4.

Sample	*n* [-]
Water	1.0
0.4% w/w CMC in water	1.0
0.8% w/w CMC in water	0.8
1% w/w CMC in water	0.6
anode slurry 10.4% w/w	1.2

**Table 5:** Transverse relaxation rates for the measured samples. Depending on the substance, either a gamma or a biexponential model was used to describe the CPMG magnetization decays. *x*_1_ and *x*_2_ represent the fractions in the biexponential model. σ represents the distribution width of the gamma distribution.

Sample Name	Transverse Relaxation Rates, Distribution Widths and Fractions
Tap water	*R*_2,mean,eff_ = 20 s^−1^ σ = 14 s^−1^
0.4% w/w CMC in water	*R*_2,1_ = 36 s^−1^, *R*_2,2_ = 7 s^−1^ *x*_1_ = 0.51, *x*_2_ = 0.49
1% w/w CMC in water	*R*_2,1_ = 37 s^−1^, *R*_2,2_ = 7 s^−1^ *x*_1_ = 0.52, *x*_2_ = 0.48
Anode slurry	*R*_2,mean,eff_ = 560 s^−1^ σ = 540 s^−1^
30% w/w graphite in water	*R*_2,mean,eff_ = 29 s^−1^ σ = 18 s^−1^
40% w/w corn starch in water	*R*_2,mean,eff_ = 83 s^−1^ σ = 71 s^−1^

## Data Availability

The data are available on request to the authors.
